# Astaxanthin Alleviates Intestinal Ferroptosis through Gut Commensal *Lepagella muris*-Mediated Retinoic Acid Production and SLC7A11 Activation

**DOI:** 10.34133/research.1421

**Published:** 2026-09-02

**Authors:** Shuqi Liu, Liang Huang, Sai Xiao, Yige Li, Shiqi Luo, Erjia Hou, Yu Zhang, Mingliang Jin, Yizhen Wang, Xin Zong

**Affiliations:** ^1^ Key Laboratory of Molecular Animal Nutrition, Ministry of Education, College of Animal Sciences, Zhejiang University, 310058 Hangzhou, PR China.; ^2^ Key Laboratory of Animal Nutrition and Feed Science in Eastern China, Ministry of Agriculture, College of Animal Sciences, Zhejiang University, 310058 Hangzhou, PR China.; ^3^ State Key Laboratory for Managing Biotic and Chemical Threats to the Quality and Safety of Agro-products, Institute of Hydrobiology, Zhejiang Academy of Agricultural Sciences, 310021 Hangzhou, PR China.; ^4^ Zhejiang Key Laboratory of Nutrition and Breeding for High-Quality Animal Products, College of Animal Sciences, Zhejiang University, 310058 Hangzhou, PR China.

## Abstract

Intestinal epithelial injury is increasingly linked to ferroptosis, yet how dietary bioactives engage the gut microbiota to restrain this process remains largely unresolved. Here, astaxanthin (ASTA) was identified as a microbiota-engaged regulator of intestinal ferroptosis and lipid peroxidation. ASTA markedly ameliorated dexamethasone-induced intestinal injury, and this protection was closely associated with the attenuation of epithelial ferroptosis. Depletion of the gut microbiota largely abolished the protective effect of ASTA, establishing the gut microbiota as an essential mediator of its intestinal bioactivity. Microbiome and metabolome profiling further revealed that ASTA reshaped the microbial metabolic landscape, with retinol metabolism emerging as a dominant pathway linked to ferroptosis resistance. Among the altered metabolites, retinoic acid was identified as a pivotal ASTA-associated metabolite that connected microbial remodeling with the restoration of epithelial anti-ferroptosis capacity. Metagenomics combined with in vitro bacterial metabolic assays identified *Lepagella muris* as a candidate ASTA-responsive bacterium capable of contributing to retinoic acid production. Mechanistically, retinoic acid protected intestinal epithelial cells from ferroptosis and barrier disruption through activation of SLC7A11, thereby reinforcing the anti-ferroptosis defense system. This study moves beyond the conventional view of ASTA as a direct antioxidant and reveals a microbiota-enabled redox metabolic mechanism that may be therapeutically exploited for ferroptosis-associated diseases.

## Introduction

Oxidative stress reflects a disruption of redox balance in which reactive oxygen species (ROS) production is no longer sufficiently buffered by endogenous antioxidant systems, a condition that contributes to the pathogenesis of cancer, neurodegenerative disorders, cardiovascular diseases, and other oxidative injury-related diseases [1–3]. Under conditions of oxidative stress, cells experience heightened ROS production, which damages proteins, lipids, and DNA, ultimately leading to cellular dysfunction and tissue injury [4]. The gut, as a vital organ involved in digestion, absorption, and immune response, is particularly susceptible to oxidative stress and its downstream effects, including inflammation and cellular death [5,6]. Once oxidative stress outstrips the cell’s antioxidant defenses, it sets off a regulated form of cell death termed ferroptosis [7]. During ferroptosis, iron-coupled lipid oxidation progressively weakens membrane integrity, particularly when iron availability is increased or anti-peroxidation defenses are impaired. These defenses include glutathione peroxidase 4 (GPX4) and the cystine/glutamate antiporter solute carrier family 7 member 11 (SLC7A11) [8,9].

Ferroptosis has been increasingly recognized as an important contributor to diverse pathological conditions, particularly those associated with tissue damage from oxidative stress and metabolic dysregulation [10–13]. When cellular antioxidant systems fail, lipid peroxidation products accumulate and trigger ferroptosis, leading to irreversible cellular damage. The key mediators of ferroptosis, including GPX4 and SLC7A11, are integral to maintaining redox homeostasis within the cell [14–16]. Given the pivotal role of oxidative stress and ferroptosis in diseases such as inflammatory bowel disease and neurodegeneration, therapeutic strategies aimed at mitigating oxidative damage and ferroptotic cell death are of great interest [17,18]. Ferroptosis is intricately linked to iron metabolism, and the accumulation of iron is a key driver of lipid peroxidation and ferroptosis [19,20]. Excessive iron catalyzes the Fenton reaction, which generates hydroxyl radicals, exacerbating oxidative damage and facilitating ferroptosis [21]. The regulation of iron homeostasis by iron-binding proteins, such as ferritin and transferrin, as well as the transporter SLC7A11, is essential for controlling ferroptosis and oxidative damage [22,23]. Astaxanthin (ASTA), a potent carotenoid found in marine organisms, has emerged as a promising candidate for combating oxidative stress [24]. Studies have shown that ASTA strengthens antioxidant capacity by enhancing enzymatic defenses such as catalase (CAT) and superoxide dismutase (SOD) while also supporting the glutathione (GSH) system [25,26]. Additionally, ASTA has been demonstrated to inhibit lipid peroxidation, reduce malondialdehyde (MDA) levels, and restore GSH levels in oxidative stress models [27], suggesting that ASTA may protect against ferroptosis.

Intestinal microbial communities participate in host metabolic regulation, immune balance, and redox-related physiological responses [28,29]. Emerging evidence indicates that the gut microbes can influence the bioavailability and biological efficacy of dietary ASTA, enhancing its potential to regulate oxidative damage [30,31]. Specifically, *Lactobacillus* and *Bifidobacterium* are commonly highlighted as beneficial bacteria that are positively influenced by ASTA. These bacteria contribute to improved gut health, metabolic balance, and reduced inflammation [32–34]. Other studies have shown that ASTA shifts the microbiota toward beneficial bacteria and restores diversity, which in turn helps alleviate oxidative stress and inflammatory damage [35]. In a high-fat diet-induced hepatic steatosis model, *Haematococcus pluvialis*-derived ASTA was linked to the enrichment of health-associated intestinal bacteria and antioxidant benefits involving the gut–liver axis [36]. These findings underscore the intricate relationship between ASTA, the gut microbiota, and oxidative stress regulation, highlighting the potential of dietary carotenoids in maintaining cellular health through microbiota-mediated mechanisms. However, how gut microbiota interacts with ASTA to regulate ferroptosis in the intestine remains unexplored.

In this study, we aim to investigate the protective effects of ASTA against dexamethasone (DEX)-induced ferroptosis and oxidative stress in intestinal cells. We focus on the role of gut microbiota in mediating ASTA metabolism to retinoic acid (RA), which may restore redox balance and modulate the ferroptosis pathway. Our findings provide insights into the molecular mechanisms by which ASTA exerts its protective effects, highlighting the potential for microbiota-mediated interventions in diseases driven by oxidative stress and ferroptosis.

## Results

### ASTA ameliorates DEX-induced intestinal injury in association with the attenuation of ferroptosis

**Fig. 1.**
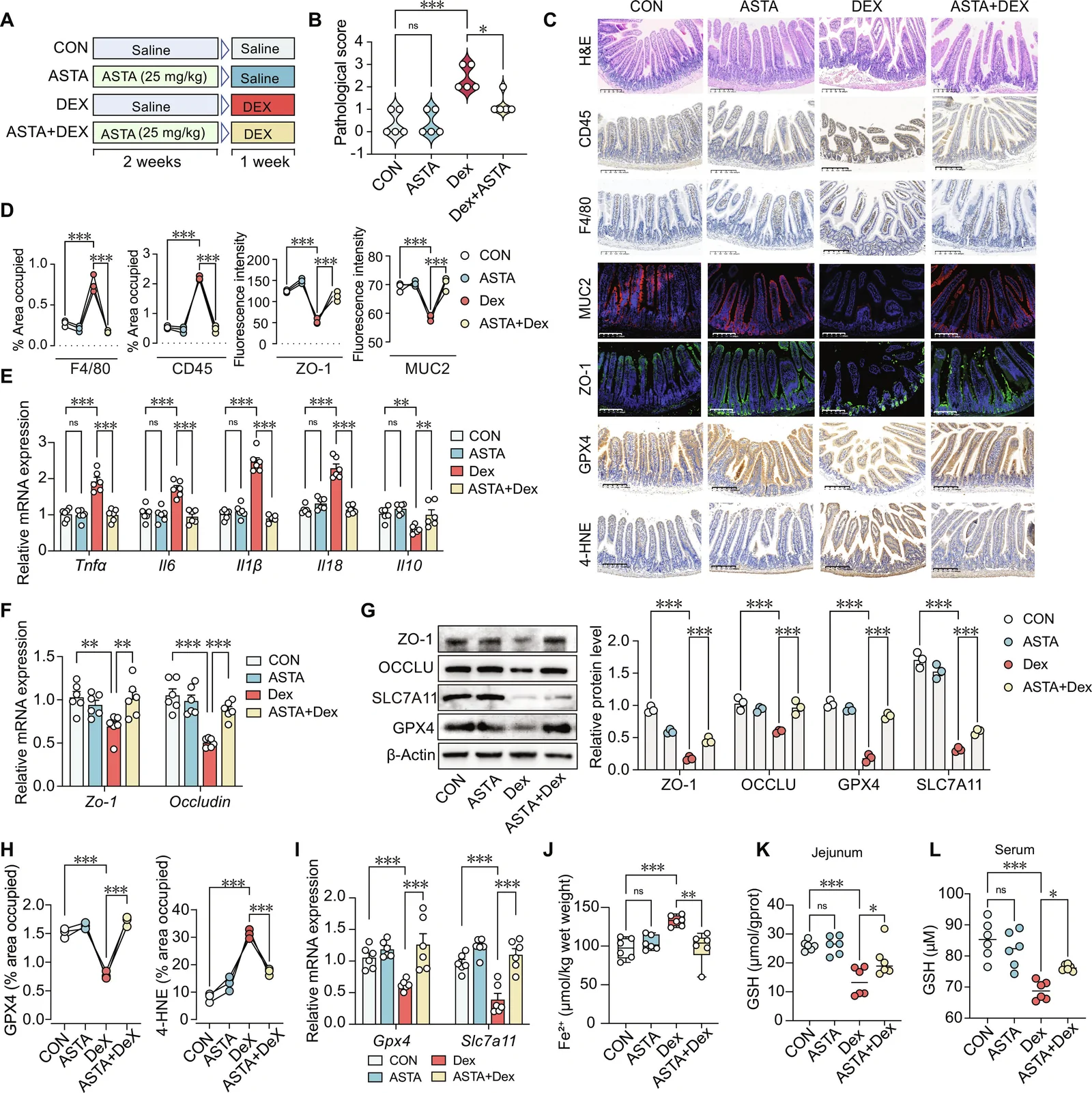
ASTA ameliorates DEX-induced intestinal injury and ferroptosis. (A) Schematic diagram of the experimental design. (B) Histopathological scores of jejunal tissues (*n* = 5). (C) Representative images of H&E staining, CD45, F4/80, 4-HNE, and GPX4 immunohistochemistry; MUC2 and ZO-1 immunofluorescence staining in jejunal sections. Scale bars, 200 μm. (D) Quantification of F4/80-positive area, CD45-positive area, and fluorescence intensity of ZO-1 and MUC2 in jejunum (*n* = 3). (E) Relative mRNA expression levels of pro-inflammatory cytokines and anti-inflammatory cytokine in jejunum, including *Tnfa*, *Il6*, *Il1β*, *Il18*, and *Il10* (*n* = 6). (F) Relative mRNA expression levels of tight junction-related genes Zo-1 and Occludin in jejunum (*n* = 6). (G) Representative Western blot images and quantitative analysis of indicated proteins in jejunum. β-Actin was used as the loading control (*n* = 3). (H) Quantification of 4-HNE and GPX4-positive area in jejunum (*n* = 3). (I) Relative mRNA expression levels of ferroptosis-related genes *Gpx4* and *Slc7a11* in jejunum (*n* = 6). (J) Fe^2+^ levels in jejunum (*n* = 6). (K and L) GSH content in jejunum (K) and serum (L) (*n* = 6). Data are presented as mean ± SEM. Each dot represents one independent biological replicate. Statistical significance was analyzed as indicated in the panels. ns, not significant; *P < 0.05; **P < 0.01; ***P < 0.001.

To determine whether ASTA protects against DEX-induced intestinal injury, a DEX-challenged mouse model was established (Fig. 1A). DEX administration caused marked disruption of jejunal architecture and significantly increased histopathological scores, whereas ASTA pretreatment notably preserved intestinal morphology and markedly reduced tissue injury, indicating a clear protective effect of ASTA against DEX-induced intestinal damage (Fig. 1B and Fig. S1A and B). We then assessed inflammatory responses and epithelial barrier integrity. DEX exposure markedly increased CD45^+^ leukocyte and F4/80^+^ macrophage infiltration, elevated the mRNA expression of pro-inflammatory cytokines, including *Tnfa*, *Il6*, *Il1β*, and *Il18*, and reduced *Il10* expression, whereas ASTA treatment alone had no significant effect on these inflammatory parameters (Fig. 1C to E). Strikingly, ASTA pretreatment significantly suppressed inflammatory cell infiltration, reduced pro-inflammatory cytokine expression, and restored *Il10* levels, indicating that ASTA effectively restrains the intestinal inflammatory response induced by DEX (Fig. 1C to E). Consistently, DEX also impaired epithelial barrier function, as reflected by reduced MUC2 and ZO-1 fluorescence intensity and reduced *Zo-1* and *Occludin* abundance at the transcript and protein levels (Fig. 1C, D, F, and G). Importantly, ASTA pretreatment markedly restored these barrier-related markers, highlighting its beneficial effect in preserving intestinal barrier integrity under DEX challenge.

Given the prominent epithelial injury, we next examined whether ferroptosis was involved in DEX-induced intestinal damage. DEX treatment markedly down-regulated *Gpx4* and *Slc7a11* expression, reduced GPX4 protein abundance and intensity, increased Fe^2+^ accumulation and enhanced 4-hydroxynonenal (4-HNE) staining in jejunal tissue, and decreased GSH levels in both the jejunum and serum, collectively indicating the induction of ferroptosis-associated lipid peroxidation (Fig. 1C, H, and I to L). In contrast, ASTA pretreatment significantly restored GPX4/SLC7A11 expression, reduced Fe^2+^ accumulation and 4-HNE deposition, and replenished GSH levels (Fig. 1C, H, and I to L). As further supporting evidence, ASTA pretreatment significantly reversed DEX-induced oxidative imbalance, reducing MDA levels in both serum and jejunal tissue while restoring CAT and SOD activities and total antioxidant capacity (T-AOC) (Fig. S2A to H).

To assess the involvement of ferroptosis in DEX-induced epithelial injury, rescue experiments were performed in IPEC-J2 cells using ferrostatin-1 (Fer-1). Similar to ASTA, Fer-1 markedly alleviated DEX-induced lipid peroxidation, mitochondrial damage, Fe^2+^ accumulation, oxidative imbalance, and ferroptosis-related gene alterations (Fig. S3A to F). These findings suggest that ASTA may protect against DEX-induced intestinal injury by suppressing inflammatory responses, preserving epithelial barrier integrity, and attenuating ferroptosis-associated oxidative lipid damage.

### Gut microbiota is required for the protective effects of ASTA against intestinal ferroptosis and barrier damage

**Fig. 2.**
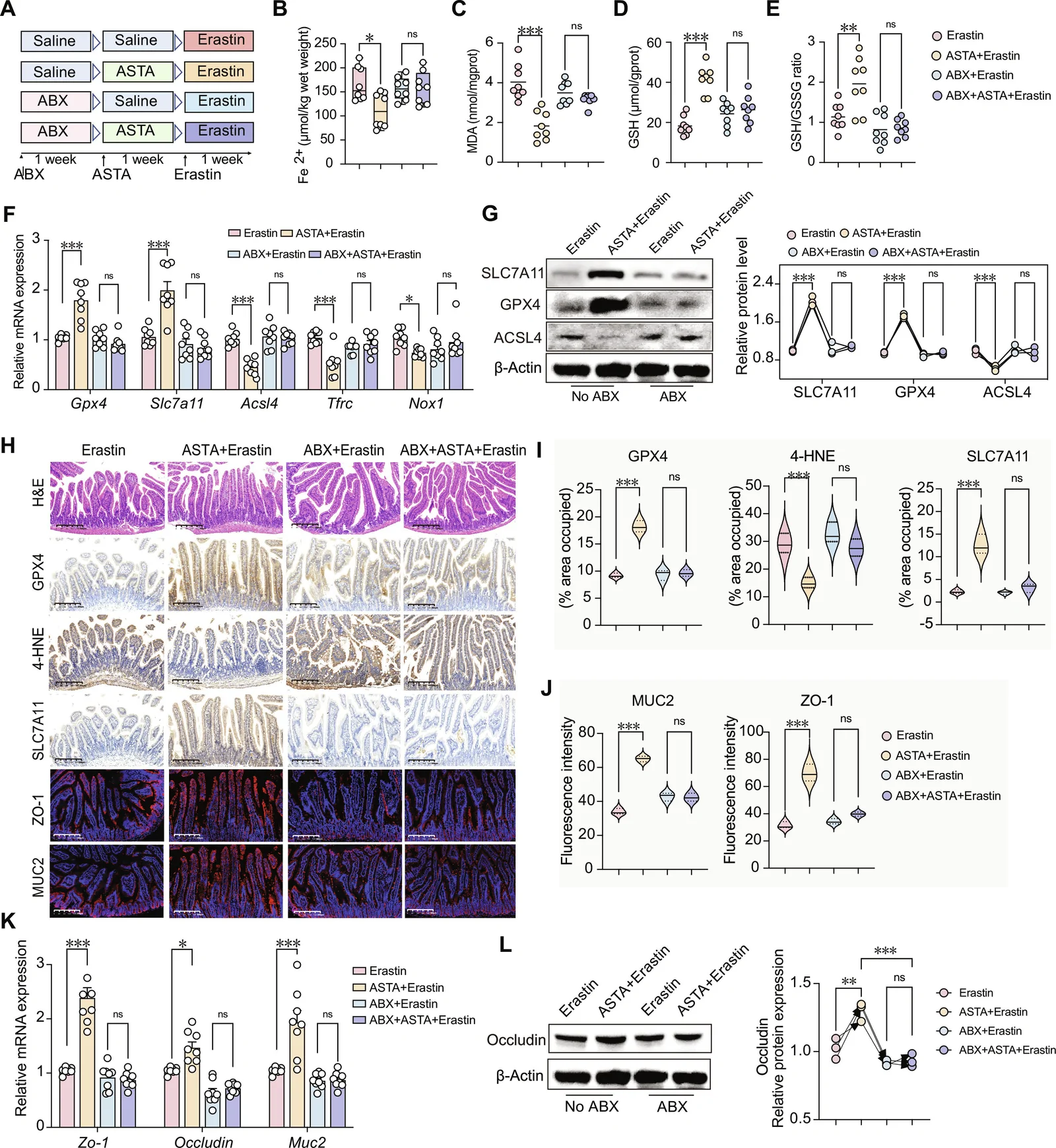
Gut microbiota depletion abolishes the protective effect of ASTA against Erastin-induced intestinal ferroptosis and barrier injury. (A) Schematic illustration of the experimental design. Mice were treated with saline or an antibiotic cocktail (ABX) for 1 week, followed by saline or ASTA for 1 week, and then challenged with Erastin for an additional week. (B to E) Biochemical analyses of ferroptosis-related parameters in intestinal tissues, including Fe^2+^ content (B), MDA levels (C), reduced glutathione (GSH) levels (D), and GSH/GSSG ratio (E) (*n* = 8). (F) Relative mRNA expression levels of ferroptosis-associated genes (*n* = 8). (G) Representative Western blot images and quantitative analysis of SLC7A11, GPX4, and ACSL4 protein expression in intestinal tissues from mice with or without ABX treatment (*n* = 3). (H) Representative images of H&E staining, immunohistochemistry for GPX4, 4-HNE, and SLC7A11, and immunofluorescence staining for ZO-1 and MUC2 in intestinal tissues from the indicated groups. Scale bars, 200 μm. (I) Quantification of the positively stained area for GPX4, 4-HNE, and SLC7A11 shown in (H) (*n* = 3). (J) Quantification of fluorescence intensity for MUC2 and ZO-1 shown in (H) (*n* = 3). (K) Relative mRNA expression levels of intestinal barrier-related genes (*n* = 8). (L) Representative Western blot images and quantitative analysis of barrier-related proteins (*n* = 3). Data are presented as mean ± SEM. Each dot represents one independent biological replicate. Statistical significance was analyzed as indicated in the panels. ns, not significant; *P < 0.05; **P < 0.01; ***P < 0.001.

Based on the ferroptosis-related protective effect of ASTA observed in the DEX model, we next used Erastin to induce a defined intestinal ferroptosis model and examined whether gut microbiota was required for ASTA-mediated protection (Fig. 2A). Under microbiota-intact conditions, ASTA markedly attenuated Erastin-induced ferroptosis alterations, as evidenced by reduced Fe^2+^ and MDA levels, restored GSH content and GSH/oxidized glutathione (GSSG) ratio, and increased expression of the ferroptosis-suppressive genes *Gpx4* and *Slc7a11* (Fig. 2B to F). Meanwhile, ASTA decreased the expression of pro-ferroptosis genes, including *Acsl4*, *Tfrc*, and *Nox1* (Fig. 2F). These findings were further confirmed at the protein and tissue levels, with ASTA increasing SLC7A11 and GPX4 expression, reducing ACSL4 abundance, and markedly limiting 4-HNE accumulation in jejunal tissues (Fig. 2G to I). However, these effects were largely abolished by antibiotic cocktail (ABX) pretreatment, as no significant differences were observed between the ABX + Erastin and ABX + ASTA + Erastin groups (Fig. 2B to I).

We next examined whether the microbiota is also required for the barrier-protective effect of ASTA using an ABX-treated mouse model, with microbiota depletion validated by a marked reduction in bacterial 16S ribosomal RNA (rRNA) gene abundance (Fig. S4). Histological assessment showed that ASTA clearly ameliorated Erastin-induced mucosal injury in the absence of ABX, whereas this protection was no longer evident following microbiota depletion (Fig. S5A to C). Consistently, ASTA significantly restored the expression of barrier-associated markers, including ZO-1, Occludin, and MUC2, as demonstrated by increased fluorescence intensity, mRNA expression, and immunostaining signals (Fig. 2H, J, K, and L). However, these restorative effects were largely lost in ABX-treated mice, and the ABX + ASTA + Erastin group displayed barrier impairment comparable to that observed in the ABX + Erastin group (Fig. 2H, J, K, and L). Together, these results indicate that the protective effect of ASTA against Erastin-induced intestinal ferroptosis and epithelial barrier damage is largely dependent on gut microbiota.

### ASTA remodels gut microbiota composition in DEX-challenged mice

**Fig. 3.**
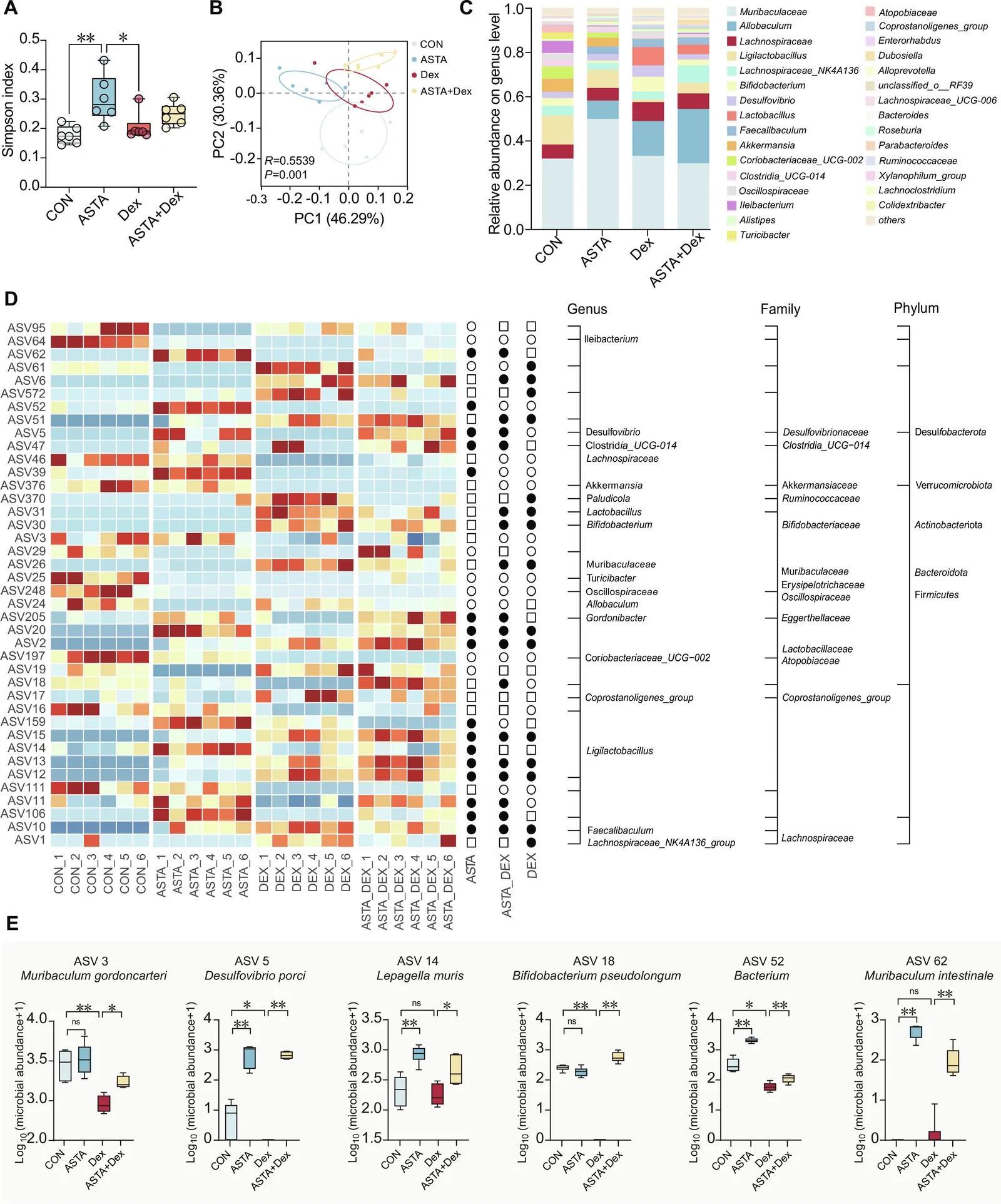
Astaxanthin modulates DEX-induced gut microbiota dysbiosis. (A) Simpson index showing the α-diversity of the gut microbiota (*n* = 6). (B) Principal coordinate analysis (PCoA) showing the β-diversity and overall microbial community structure among groups. The degree of group separation is indicated by the analysis of similarities (ANOSIM) statistics shown in the plot (*n* = 6). (C) Relative abundance of the dominant bacterial genera in each group. (D) Heatmap showing the abundance patterns of representative differential amplicon sequence variants (ASVs) across individual samples, together with the taxonomic annotation of group-associated ASVs at the genus, family, and phylum levels. (E) Boxplot analysis of selected discriminatory ASVs, including ASV3 (*M. gordoncarteri*), ASV5 (*D. porci*), ASV14 (*L. muris*), ASV18 (*B. pseudolongum*), ASV52 (*Bacterium*), and ASV62 (*M. intestinale*).

Given that gut microbiota depletion largely abolished the protective effect of ASTA against intestinal ferroptosis injury, we next performed 16S rRNA sequencing to determine whether ASTA reshapes the gut microbial community under DEX challenge. Analysis of α-diversity showed that ASTA significantly increased the Simpson index compared with the control group, whereas the DEX group displayed a lower diversity profile than the ASTA group (Fig. 3A). In parallel, principal coordinate analysis (PCoA) revealed a clear separation of microbial communities among the 4 groups, indicating that both DEX exposure and ASTA intervention markedly altered the overall gut microbial structure (Fig. 3B). These findings suggest that ASTA supplementation is associated with a distinct remodeling of the intestinal microbiota in DEX-treated mice.

At the taxonomic level, phylum and genus composition analysis showed that DEX challenge induced an evident redistribution of dominant bacterial taxa, whereas ASTA cotreatment partially shifted the microbial profile away from that observed in the DEX group (Fig. 3C and Fig. S6A to E). Differential abundance analysis further identified distinct taxonomic signatures across groups. Notably, ASTA significantly reduced the abundance of pathogenic taxa such as *Desulfovibrio* while increasing the relative abundance of beneficial taxa like *Akkermansia*, *Bacteroides*, *Ligilactobacillus*, and *Faecalibaculum* (Fig. 3D)*.* At the amplicon sequence variant (ASV) level, ASTA treatment reversed the DEX-induced decrease in *Muribaculum gordoncarteri* (ASV 3), *Desulfovibrio porci* (ASV 5), *Lepagella muris* (ASV 14), *Bifidobacterium pseudolongum* (ASV 18), *Bacterium* (ASV 52), and *Muribaculum intestinale* (ASV 62), with these taxa showing significant increases in the ASTA-treated groups (Fig. 3E), further supporting a pronounced microbiota-modulating effect of ASTA. Correlation analysis revealed that the abundance of these bacteria was positively correlated with increased levels of T-AOC while inversely correlating with inflammatory and ferroptosis markers, including *Il1β* gene expression and Fe and Fe^2+^ concentration (Fig. S7A to C). Collectively, these results indicate that ASTA markedly remodels the gut microbial community in DEX-challenged mice and partially reverses DEX-associated microbial dysbiosis, supporting a close association between microbiota reprogramming and the intestinal protective effect of ASTA.

### Gut microbiota mediates ASTA-associated metabolic reprogramming involving retinol metabolism and RA

**Fig. 4.**
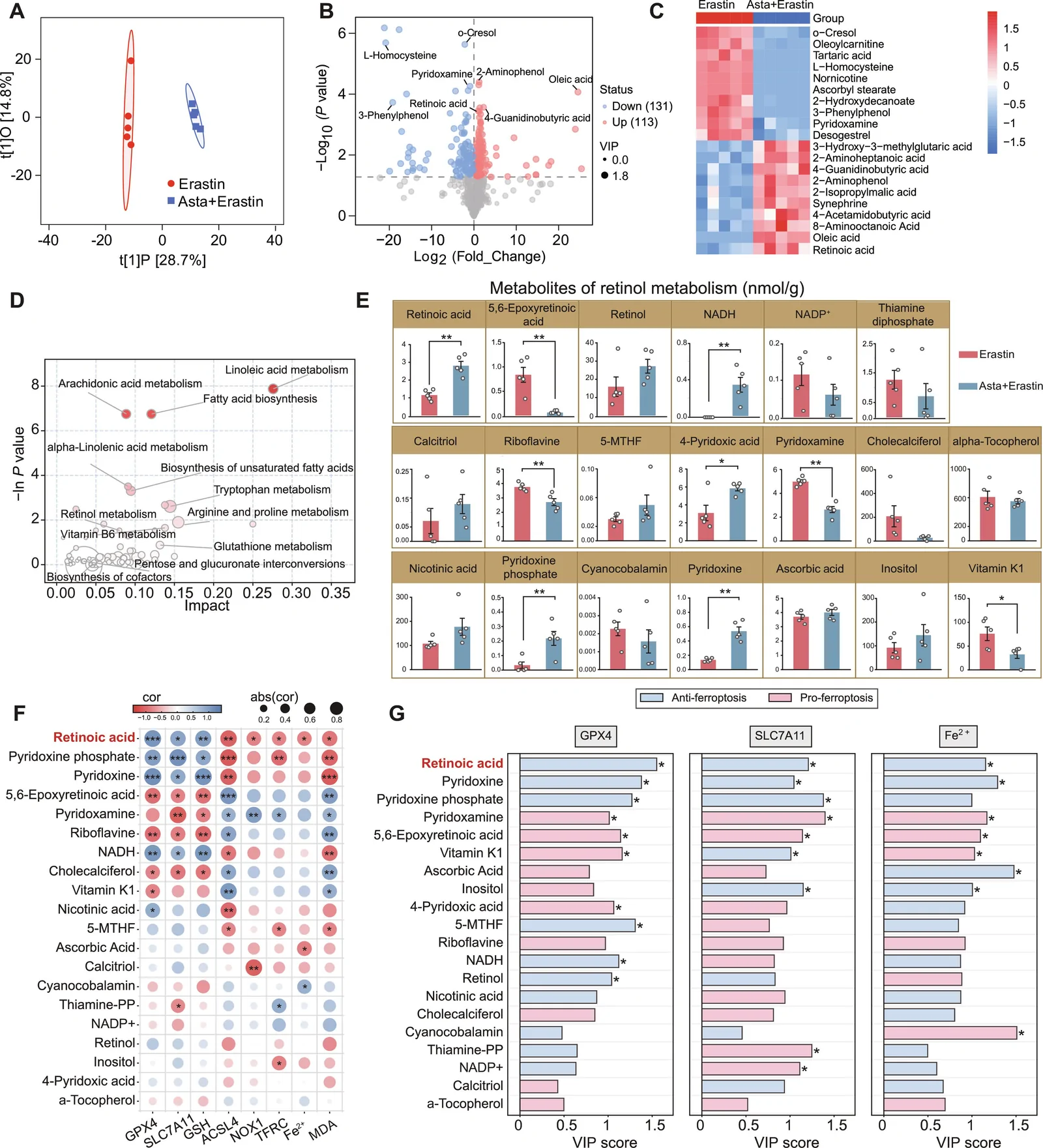
Metabolomic profiling identifies retinoic acid-associated metabolic reprogramming in ASTA-mediated protection against Erastin-induced ferroptosis. (A) OPLS-DA score plot showing the overall separation of metabolomic profiles. (B) Volcano plot of differential metabolites between the Erastin and ASTA + Erastin groups. Red dots indicate metabolites up-regulated in the ASTA + Erastin group, blue dots indicate down-regulated metabolites, and gray dots indicate unchanged metabolites. Point size represents the variable importance in projection (VIP) score. (C) Heatmap showing the relative abundance of representative differential metabolites between the 2 groups. (D) Pathway enrichment and topology analysis of differential metabolites. (E) Quantitative analysis of metabolites related to retinol metabolism and associated vitamin/cofactor metabolic pathways. (F) Correlation matrix showing the associations between selected metabolites and ferroptosis-related indices, including *Gpx4*, *Slc7a11*, GSH, *Acsl4*, *Nox1*, *Tfrc*, Fe^2+^, and MDA. Color represents the correlation coefficient, and dot size represents the absolute correlation strength. (G) VIP scores of metabolites associated with anti-ferroptosis and pro-ferroptosis signatures based on *Gpx4*, *Slc7a11*, and Fe^2+^-related models. Data in (E) are presented as mean ± SEM from *n* = 5 biological replicates. Each dot represents one independent biological replicate. Statistical significance was analyzed as indicated in the panels. ns, not significant; *P < 0.05; **P < 0.01; ***P < 0.001.

To further investigate the role of gut microbiota in mediating the beneficial effects of ASTA on intestinal ferroptosis, targeted metabolomics was performed under Erastin challenge. Orthogonal projection to latent structures–discriminant analysis (OPLS-DA) showed a clear separation between the Erastin and ASTA + Erastin groups (Fig. 4A), indicating that ASTA markedly reshaped the metabolic profile under ferroptosis stress. Consistently, volcano plot analysis identified 113 up-regulated and 131 down-regulated metabolites in the ASTA + Erastin group (Fig. 4B). Hierarchical clustering further revealed a distinct metabolic signature after ASTA treatment (Fig. 4C). These results indicate that ASTA induces pronounced metabolic reprogramming during ferroptosis injury.

We next analyzed the metabolic pathways altered by ASTA. Pathway enrichment showed that the differential metabolites were mainly involved in linoleic acid metabolism, arachidonic acid metabolism, fatty acid biosynthesis, biosynthesis of unsaturated fatty acids, vitamin B6 metabolism, glutathione metabolism, and retinol metabolism (Fig. 4D). Given the close relationship between ASTA, retinoid metabolism, intestinal homeostasis, and redox regulation, retinol metabolism was selected for further investigation. As shown in Fig. 4E, ASTA significantly increased RA levels while reducing 5,6-epoxyretinoic acid. ASTA also altered several vitamin-related metabolites, including pyridoxine, pyridoxine phosphate, and pyridoxamine, indicating coordinated remodeling of redox-related metabolic networks.

Additionally, to assess the relationship between these metabolites and ferroptosis, correlation analysis was performed with ferroptosis-related indices. RA was positively correlated with the expression of the anti-ferroptosis markers *Gpx4* and *Slc7a11*, as well as GSH levels (Fig. 4F). In contrast, it was negatively correlated with the expression of the pro-ferroptosis markers *Acsl4*, *Nox1*, and *Tfrc*, and with Fe^2+^ and MDA levels (Fig. 4F). Variable importance analysis further highlighted RA as a major metabolite contributing to the changes in GPX4 and SLC7A11 expression and Fe^2+^ levels (Fig. 4G). Taken together, these findings suggest that ASTA induces gut microbiota-associated metabolic reprogramming under ferroptosis stress. Retinol metabolism may represent a potential downstream metabolic axis, and RA may serve as a key metabolite associated with the protective effect of ASTA against intestinal ferroptosis.

### Gut microbe *L. muris* contributes to ferroptosis alleviation by mediating the conversion of ASTA to RA

**Fig. 5.**
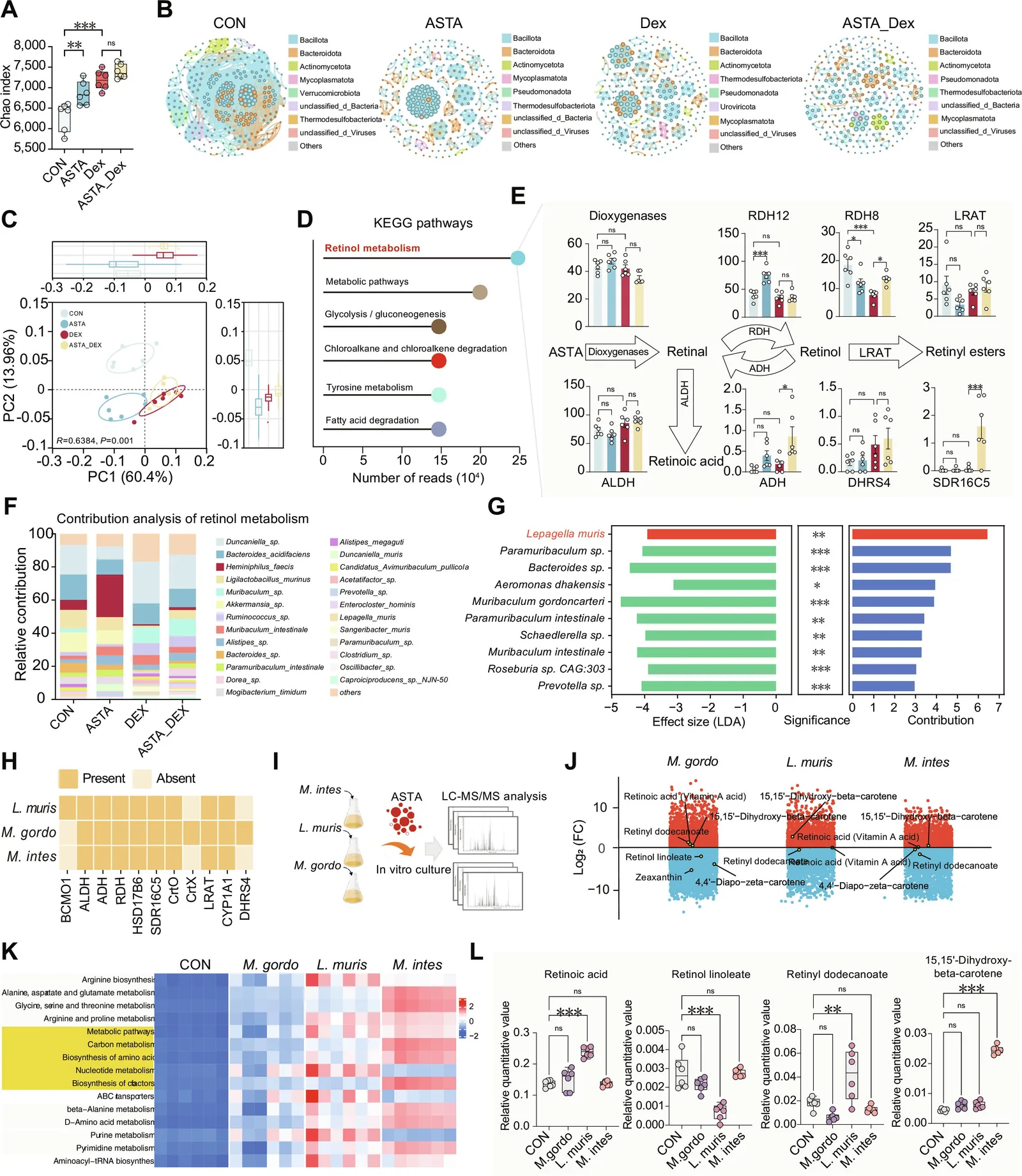
ASTA remodels gut retinol metabolism and identifies retinoic acid produced by candidate bacteria. (A) Alpha-diversity of the gut microbiota shown by the Chao1 index (*n* = 6). (B) Global microbial co-occurrence network and group-specific ecological networks colored by phylum-level taxonomy. (C) Principal coordinate analysis (PCoA) of microbial community structure; the corresponding marginal distributions are shown above and on the right (*n* = 6). (D) KEGG pathway analysis of metagenomic reads, highlighting retinol metabolism as a prominently enriched pathway. (E) Schematic overview of the retinol metabolic pathway and relative abundance of retinol metabolism-related enzymes, including dioxygenases, RDH12, RDH8, LRAT, ALDH, ADH, DHRS4, and SDR16C5 (*n* = 6). (F) Relative contribution of individual bacterial taxa to the retinol metabolism pathway. (G) Key taxa associated with retinol metabolism identified by linear discriminant analysis (LDA) effect size, significance, and contribution analyses. (H) Heatmap illustrating the presence or absence of key retinol metabolism-related enzymes in the genomes of *L. muris*, *M. gordoncarteri*, and *M. intestinale*. (I) Schematic diagram depicting the experimental setup for metabolic analysis of ASTA in *L. muris*, *M. gordoncarteri*, and *M. intestinale*. (J) Volcano plot highlighting the significant metabolites identified in the metabolic profiling of ASTA-treated strains. (K) Heatmap of pathway enrichment across control samples and the 3 candidate bacterial cultures. (L) Relative quantification of representative retinoid-related metabolites, including retinoic acid, retinol linoleate, retinyl dodecanoate, and 15,15′-dihydroxy-β-carotene, in control and bacterial culture groups (*n* = 6). Data are presented as box plots or mean ± SEM with individual data points shown where indicated. Statistical significance was analyzed as indicated in the panels. ns, not significant; *P < 0.05; **P < 0.01; ***P < 0.001.

Subsequently, to further investigate how gut microbiota contributes to the alleviation of DEX-induced ferroptosis by ASTA, metagenomic sequencing was performed. Microbial richness analysis showed that both DEX and ASTA altered the overall community structure, as reflected by changes in the Chao index (Fig. 5A). Taxonomic level and community network analysis further revealed marked differences in microbial organization among the 4 groups (Fig. 5B and Fig. S9A to D). Consistently, PCoA showed a clear separation of microbial communities, indicating that DEX challenge markedly disturbed gut microbial structure and that ASTA intervention reshaped this profile (Fig. 5C). These findings further confirm that ASTA exerts a substantial modulatory effect on gut microbiota under DEX treatment.

We next examined the functional changes in the gut microbiome. Kyoto Encyclopedia of Genes and Genomes (KEGG) pathway annotation revealed that retinol metabolism was among the most prominent ASTA-responsive microbial pathways (Fig. 5D). Analysis of key enzymes involved in retinol metabolism showed that most upstream carotenoid-cleaving dioxygenases and aldehyde dehydrogenase (ALDH) were not markedly altered among groups, whereas alcohol dehydrogenase (ADH) showed a notable increase in the ASTA + DEX group relative to the DEX group (Fig. 5E). These data suggest that ASTA may be associated with enhanced conversion of retinol to retinal within the retinoid pathway. Consistently, contribution analysis further showed that ASTA markedly altered the microbial contributors to the retinol metabolism pathway (Fig. 5F). To identify the taxa potentially involved in this process, differential microbial analysis was performed. Several ASTA-responsive species were enriched within the retinol metabolism-associated microbiota, including *L. muris*, *M. gordoncarteri*, and *M. intestinale* (Fig. 5G). Among these candidates, *L. muris* showed a prominent contribution to the retinol metabolism-associated microbial signature. We therefore performed a comprehensive genome-wide analysis of *M. gordoncarteri*, *L. muris*, and *M. intestinale*, with a specific focus on genes involved in retinol metabolism (Fig. S10). Notably, although ALDH was detected in all 3 bacterial species, only *L. muris* harbored β-carotene 15,15′-monooxygenase 1 (BCMO1)-like carotenoid-cleaving dioxygenases, thereby providing a relatively complete enzymatic repertoire for the conversion of ASTA to RA and suggesting a more central role for *L. muris* in RA biosynthesis (Fig. 5H).

To validate this prediction, in vitro metabolic assays were performed by incubating ASTA with *M. gordoncarteri*, *L. muris*, and *M. intestinale*, followed by liquid chromatography–tandem mass spectrometry (LC-MS/MS) analysis (Fig. 5I). Metabolomic profiling showed that these bacteria exhibited distinct retinoid metabolic patterns after ASTA exposure (Fig. 5J and K). Importantly, *L. muris* significantly increased RA production compared with the control condition, whereas *M. gordoncarteri* showed no obvious effect (Fig. 5L). In contrast, *M. intestinale* was more strongly associated with the accumulation of 15,15′-dihydroxy-β-carotene, suggesting a tendency to retain upstream carotenoid-derived intermediates rather than efficiently generate RA (Fig. 5L). *L. muris* also altered other retinoid-related metabolites, including retinol linoleate and retinyl dodecanoate, further supporting its active role in retinoid conversion. Together, these findings indicate that ASTA reshapes the structure and functional potential of the gut microbiota under DEX challenge. This remodeling is closely associated with retinol metabolism. Among the candidate bacteria, *L. muris* appears to be a key microbe linking ASTA intervention to enhanced retinoid metabolism, potentially by facilitating the bioconversion of ASTA toward RA.

### RA reduces Erastin-induced ferroptosis and epithelial barrier injury through SLC7A11 activation

**Fig. 6.**
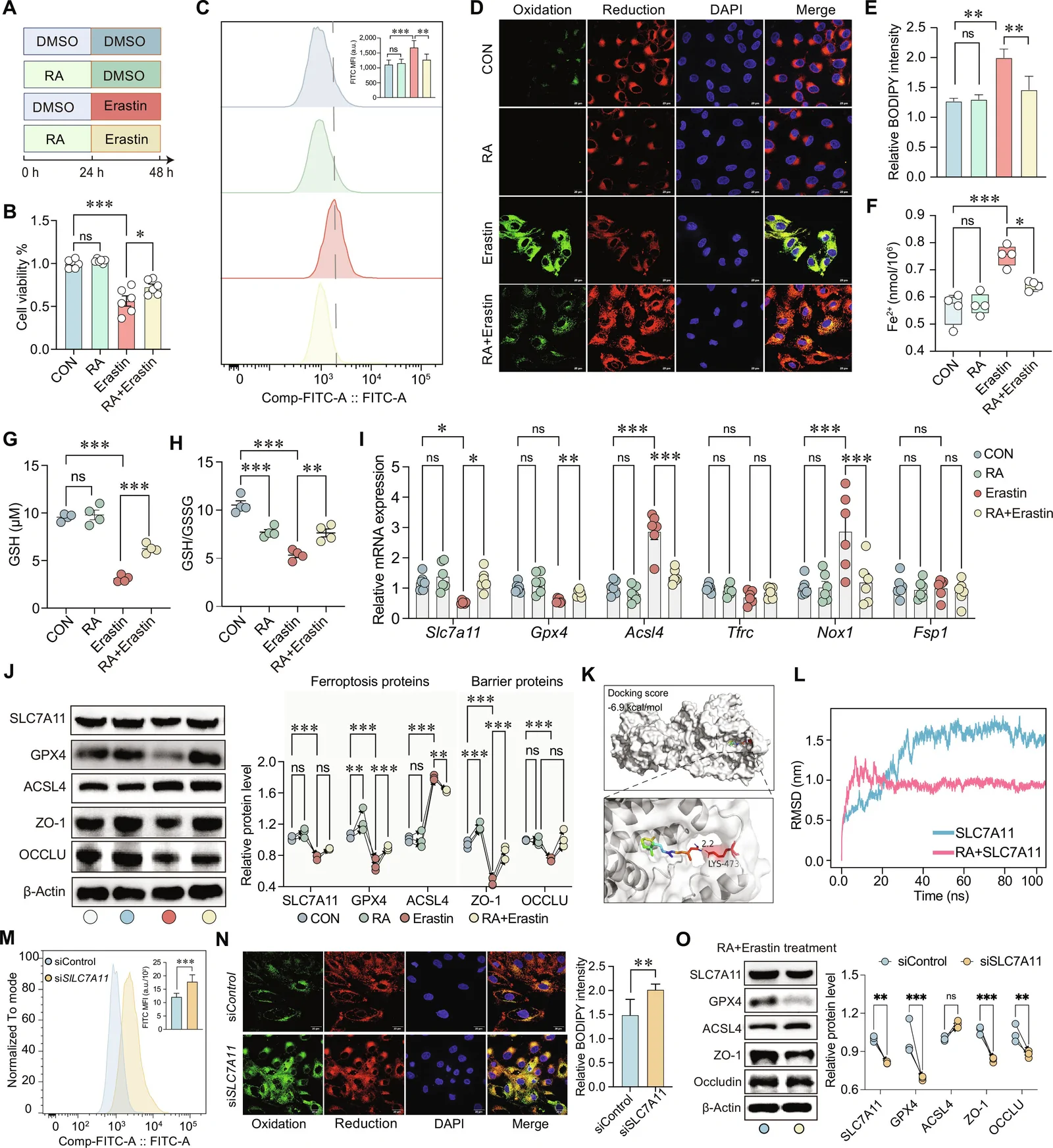
SLC7A11 mediates the protective effect of retinoic acid against Erastin-induced ferroptosis in intestinal epithelial cells. (A) Treatment scheme. (B) Cell viability (*n* = 6). (C to E) Lipid peroxidation assessed by BODIPY 581/591 C11 staining and analyzed by flow cytometry (C) and confocal microscopy (D) with corresponding quantification in (E); scale bars, 50 μm (*n* = 3). (F to H) Intracellular Fe^2+^ (F), GSH (G) and GSH/GSSG (H) levels (*n* = 4). (I) mRNA expression of *SLC7A11*, *GPX4*, *ACSL4*, *TFRC*, *NOX1*, and *FSP1* (*n* = 4). (J) Western blot and quantification of ferroptosis-related (SLC7A11, GPX4, ACSL4) and barrier-related (ZO-1, Occludin) proteins; β-actin as loading control (*n* = 3). (K and L) Molecular docking pose (K) and RMSD trajectory (L) of the RA-SLC7A11 complex. (M and N) BODIPY 581/591 C11 staining analyzed by flow cytometry (M) and confocal microscopy (N) with quantification in IPEC-J2 cells transfected with siControl or si*SLC7A11* under RA + Erastin treatment; scale bars, 50 μm (*n* = 3). (O) Western blot and quantification of the indicated proteins under the same conditions (*n* = 3). Data are mean ± SEM. ns, not significant; *P < 0.05; **P < 0.01; ***P < 0.001.

To investigate the protective effects of RA on ferroptosis, we established an intestinal epithelial cell model using Erastin (Fig. 6A). Notably, Erastin markedly reduced cell survival, whereas RA pretreatment significantly restored viability under Erastin exposure (Fig. 6B). Consistent with a protective role against ferroptosis, RA substantially suppressed Erastin-induced lipid peroxidation, as shown by reduced oxidized BODIPY 581/591 C11 signals in both flow cytometric and confocal analyses (Fig. 6C to E). RA also limited ferrous iron accumulation and preserved intracellular GSH homeostasis, further supporting its ability to restrain ferroptosis stress (Fig. 6F to H), accompanied by reduced intracellular oxidant levels (Fig. S11A). Moreover, RA reversed the Erastin-induced ferroptosis program by restoring the expression of *SLC7A11* and *GPX4* while suppressing *ACSL4* and *NOX1*, with no obvious regulatory effect on *TFRC* or *FSP1* (Fig. 6I and J). In parallel, RA preserved epithelial barrier integrity, as reflected by restored ZO-1 and Occludin expression under Erastin challenge (Fig. 6J and Fig. S11B). These findings provide in vitro evidence that RA strengthens intestinal epithelial barrier integrity through the suppression of ferroptosis.

To explore whether SLC7A11 serves as a potential functional node mediating this protective effect, we performed molecular docking analysis, which predicted a possible interaction between RA and SLC7A11, with a docking score of −6.9 kcal/mol, and the predicted binding was further supported by molecular dynamics simulations showing enhanced structural stability of the RA-SLC7A11 complex (Fig. 6K and L and Fig. S13A). To further assess the functional involvement of SLC7A11, *SLC7A11* was silenced using small interfering RNA (siRNA) (Fig. S12). Notably, in RA pretreated cells challenged with Erastin, *SLC7A11* knockdown substantially diminished the protective effect of RA, as shown by markedly enhanced lipid peroxidation, as evidenced by an increased mean fluorescence intensity of oxidized BODIPY and an elevated oxidized/reduced BODIPY ratio (Fig. 6M and N). Consistently, RA-mediated ferroptosis resistance was largely reversed after SLC7A11 knockdown, as shown by decreased SLC7A11 and GPX4 expression, increased ACSL4 expression, and re-accumulation of intracellular oxidants (Fig. 6O and Fig. S13B and C). In parallel, the barrier-preserving effect of RA was also impaired, evidenced by reduced ZO-1 and Occludin expression (Fig. 6O and Fig. S13D). Together, these data demonstrate that SLC7A11 is indispensable for RA-mediated protection against ferroptosis-related epithelial barrier disruption.

## Discussion

The present study identifies a microbiota-associated RA metabolic axis that may contribute to ASTA-mediated intestinal protection. Rather than acting solely as a direct antioxidant, ASTA appears to engage a host–microbiota metabolic circuit that strengthens epithelial anti-ferroptosis defense. This metabolic remodeling was associated with enhanced RA-related metabolism, restoration of epithelial defense pathways, and attenuation of lipid peroxidative injury.

ASTA has long been recognized as a potent dietary carotenoid with antioxidant and anti-inflammatory properties [37,38]. In the context of intestinal injury, however, the protective activity of carotenoids is unlikely to be explained only by chemical scavenging of ROS. The intestinal lumen is a metabolically active interface where dietary compounds are transformed by resident microbes before influencing host epithelial responses [39]. In this study, ASTA markedly attenuated multiple ferroptosis-related alterations induced by DEX, including iron accumulation, lipid peroxidation, and suppression of the SLC7A11/GPX4 pathway, while the Erastin challenge model and Fer-1 rescue experiments further supported the involvement of ferroptosis in DEX-induced intestinal injury. The DEX and Erastin models were used for complementary purposes, with DEX employed to assess intestinal injury and ASTA-associated microbial and metabolic changes, and Erastin used to provide a defined ferroptosis challenge for evaluating microbiota dependence. These findings extend the conventional view of ASTA as an antioxidant molecule by showing that its protective effect is closely connected to ferroptosis suppression. These observations support ferroptosis suppression as an important component of ASTA-mediated intestinal protection and suggest that targeting epithelial redox-lipid metabolism may be an effective strategy for preserving intestinal barrier integrity under stress conditions.

Accumulating evidence indicates that specific gut microbial taxa can shape host nutrient metabolism, mucosal immunity, and epithelial homeostasis [40–42]. In this context, our ABX experiments provide evidence that the protective effect of ASTA against intestinal ferroptosis is at least partly microbiota dependent. Although broad-spectrum antibiotics indiscriminately deplete both beneficial and potentially detrimental microbial taxa, ABX treatment alone did not significantly aggravate Erastin-induced ferroptosis injury under the conditions examined in this study. By contrast, microbiota depletion markedly diminished the ability of ASTA to restore ferroptosis-related intestinal injury. These findings suggest that microbiota depletion does not independently promote ferroptosis under the conditions examined, but that the protective effect of ASTA is at least partly dependent on the gut microbiota.

The biological activity of dietary carotenoids is increasingly understood to depend not only on host absorption and metabolism but also on microbial transformation within the gut [43,44]. Our metagenomic and metabolomic analyses suggest that ASTA alters the microbial metabolic landscape in a manner associated with enhanced RA biosynthesis. This finding shifts the interpretation of ASTA action from a host-centered antioxidant model to a microbiota-linked metabolic model, in which microbial communities help convert dietary carotenoid signals into host-protective metabolites. Among the microbial changes induced by ASTA, *L. muris* was associated with RA-related metabolic activity and may represent a candidate microbial contributor to this pathway, providing a plausible microbial link between ASTA and retinoid-associated metabolic remodeling. *L. muris* is a member of the family *Muribaculaceae*, a group of bacteria prevalent in the murine gut that are increasingly recognized for their roles in polysaccharide degradation and host–microbe metabolic interactions [45]. The predicted presence of BCMO1-like and ALDH enzymes in *L. muris* suggests a potential capacity to participate in carotenoid-associated retinoid metabolism. The recognition of *L. muris* as a pivotal microbe involved in RA synthesis also raises important questions about its potential therapeutic applications. This discovery is consistent with recent studies that have explored the capacity of gut microbiota to produce active retinoids and influence host immune responses and metabolic processes [46–48]. Nevertheless, this association should be interpreted cautiously. Direct evidence from targeted colonization, bacterial metabolite production assays, or fecal microbiota transplantation will be required to determine whether *L. muris* is a causal driver of RA production or part of a broader microbial network that supports carotenoid-derived retinoid metabolism.

Retinoid signaling is well known to regulate mucosal immunity, epithelial differentiation, and barrier maintenance [49–51]. Our results indicate that RA is associated with up-regulation of SLC7A11, a cystine/glutamate antiporter important for maintaining cellular glutathione levels [51]. Increased SLC7A11 expression may support GPX4 activity and facilitate lipid peroxide detoxification. Thus, RA may function not only as an immunoregulatory metabolite but also as a metabolic signal that strengthens epithelial anti-ferroptosis defense. SLC7A11 may represent a candidate downstream effector linking RA signaling to ferroptosis resistance. The restoration of SLC7A11 and GPX4 by ASTA and RA suggests that this pathway may contribute importantly to limit ferroptosis-related epithelial injury.

**Fig. 7.**
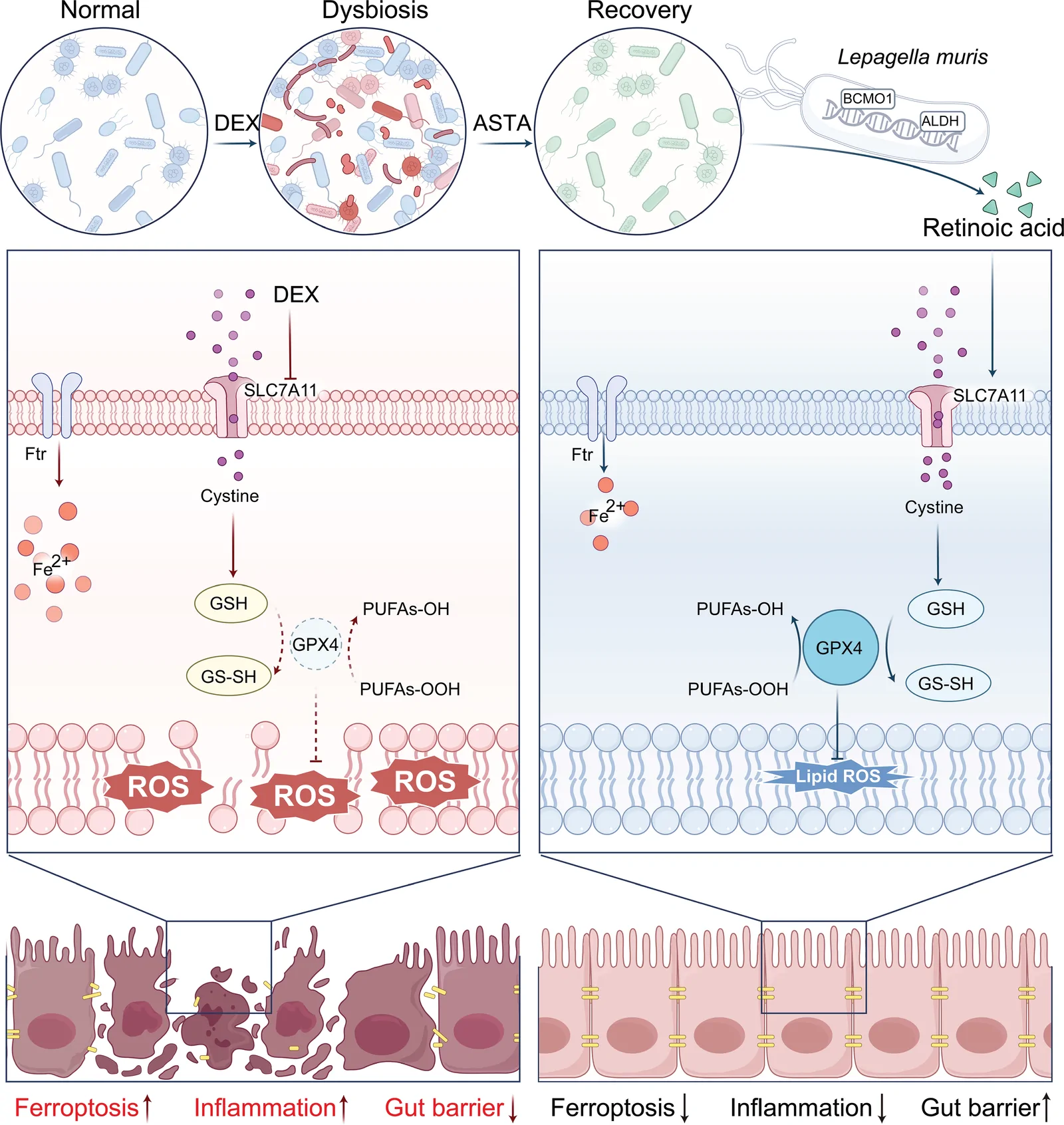
Proposed model of ASTA-mediated intestinal protection through a microbiota-associated retinoic acid–SLC7A11 axis. ASTA attenuates intestinal lipid peroxidation and remodels the gut microbial metabolic landscape. *Lepagella muris* is proposed as an ASTA-responsive candidate microbial contributor with retinoid-metabolic potential that may facilitate retinoic acid (RA) production. RA promotes SLC7A11/GPX4-dependent antioxidant defense in intestinal epithelial cells, thereby limiting lipid peroxidation and ferroptosis-related epithelial injury and preserving epithelial barrier integrity.

Interestingly, previous studies have suggested that ASTA can act directly on intestinal epithelial cells by limiting intracellular lipid peroxidation. Consistent with these observations, our in vitro data showed that ASTA directly attenuated lipid peroxidation and ferroptosis-related injury in intestinal epithelial cells. However, this cell-autonomous effect alone does not fully explain the microbiota-dependent protection observed in vivo, as depletion of the gut microbiota markedly diminished the protective efficacy of ASTA. Meanwhile, RA enhanced SLC7A11-associated antioxidant defense in intestinal epithelial cells, supporting a role for microbiota-associated retinoid metabolism in reinforcing epithelial resistance to ferroptosis. We therefore propose that the direct antioxidant activity of ASTA and the microbiota-associated RA-SLC7A11 signaling axis represent complementary, rather than mutually exclusive, mechanisms that collectively contribute to its intestinal protective effects. Conceptually, these findings support a working model in which ASTA remodels gut microbial metabolism, microbiota-associated RA production is enhanced, RA promotes SLC7A11/GPX4-dependent antioxidant defense, and epithelial ferroptosis is consequently suppressed (Fig. 7). These findings also suggest that microbial or metabolite-targeted strategies may enhance the intestinal benefits of dietary carotenoids. Despite these findings, several limitations should be acknowledged. Although RA was identified as a key metabolite associated with ASTA-mediated protection, its causal role in vivo remains to be established. In addition, apoptosis, pyroptosis, and necroptosis were not systematically examined, and their possible contributions to DEX-induced epithelial injury cannot be excluded. Moreover, because this study was conducted primarily in mouse models and intestinal epithelial cells, validation in human samples and clinically relevant models will be necessary to determine the translational relevance of this mechanism, which may benefit from emerging precision medicine and computational approaches for therapeutic prediction [52,53].

## Conclusion

In conclusion, our study identifies a previously underappreciated mechanism through which ASTA may protect the intestine from oxidative stress and ferroptosis-related injury. This protection is linked to gut microbiota-mediated RA biosynthesis and downstream activation of SLC7A11. These findings place dietary carotenoid metabolism within a broader diet–microbiota–host framework for maintaining intestinal health. They also provide a mechanistic basis for developing microbiota-targeted strategies to limit ferroptosis-related intestinal injury.

## Materials and Methods

### Reagents

All reagents, antibodies, commercial kits, and biological resources used in this study, together with their sources, are listed in Table S1.

### Animal model

Male C57BL/6 mice aged 6 to 8 weeks were sourced from SLAC Laboratory Animal Co. Ltd. and housed in an animal room maintained on a 12-h light/12-h dark schedule. After a 1-week acclimatization period, the mice were randomly assigned to one of 4 groups (*n* = 6 per group): control (vehicle), DEX, ASTA + DEX, and ASTA. DEX (15 mg/kg) was administered via intraperitoneal injection for 7 consecutive days. In the ASTA + DEX group, 25 mg/kg ASTA was administered by oral gavage for 2 weeks prior to DEX treatment. The control group received phosphate-buffered saline (PBS) as the vehicle. On day 22, jejunal samples were collected after euthanasia for histology, biochemical assays, and gene-expression analysis.

To determine whether gut microbiota were required for ASTA-mediated protection in the Erastin-induced intestinal ferroptosis model, mice were allocated to the Erastin, ASTA + Erastin, an antibiotic cocktail (ABX) + Erastin, and ABX + ASTA + Erastin groups. For microbiota depletion, mice received an antibiotic cocktail continuously for 1 week via drinking water. The cocktail contained ampicillin, metronidazole, vancomycin, and neomycin, each at 0.5 g/l, and was freshly prepared every 2 d. Mice had free access to the antibiotic-containing water throughout the depletion period. The efficiency of microbiota depletion was evaluated by quantitative real-time polymerase chain reaction (qPCR) targeting the bacterial 16S rRNA gene in fecal DNA collected from the same mice before and after antibiotic treatment. ASTA was administered by oral gavage at 25 mg/kg/day for 1 week. Erastin was then administered at 30 mg/kg by intraperitoneal injection for 1 week to induce intestinal ferroptosis and barrier injury. Control animals received an equal volume of saline as appropriate. After the final treatment, mice were euthanized and intestinal tissues were sampled.

### Cell culture

Porcine intestinal epithelial IPEC-J2 cells were maintained in Dulbecco’s modified Eagle’s medium (DMEM)/F-12 supplemented with 10% fetal bovine serum and 1% penicillin–streptomycin at 37 °C in 5% CO_2_, as previously described [34].

For the DEX experiment, cells were assigned to the control, Fer-1, DEX, Fer-1 + DEX, and ASTA + DEX groups. Cells were pretreated with 2 μM Fer-1 or 20 μM ASTA for 2 h, followed by 1 μM DEX exposure in the continued presence of Fer-1 or ASTA for 24 h. Lipid peroxidation, MDA, intracellular Fe^2+^, GSH/GSSG, ferroptosis-related gene expression, and mitochondrial ultrastructure were subsequently assessed.

For the RA experiment, cells were pretreated with 1 μM RA for 24 h and then exposed to 10 μM Erastin for another 24 h. Cell viability was determined using the Cell Counting Kit-8 assay. Vehicle-treated cells served as controls.

### Histological analysis

Jejunal histology was evaluated following the protocol reported in Ref. [54]. After euthanasia, jejunal segments were excised, fixed in 10% neutral-buffered formalin for 24 h, and processed for paraffin embedding. The tissue was sectioned into 5-μm-thick slices using a rotary microtome (Leica RM2235, Leica Microsystems, Germany). After dewaxing in xylene and rehydration in a graded ethanol series, sections were stained with hematoxylin and eosin (H&E) for routine histopathology. To assess intestinal integrity and cellular morphology, the stained sections were examined under a light microscope (Olympus IX71, Olympus Corporation, Japan). Images were captured and analyzed using a digital camera system (Olympus DP72). Quantification of villus height, crypt depth, and villus/crypt ratio was performed using ImageJ software (version 1.54), with at least 5 fields of view per sample.

Histopathological injury of the jejunum was evaluated in a blinded manner using a semiquantitative 0 to 4 scoring system [55]. A score of 0 indicated normal mucosal architecture without obvious injury; 1 indicated mild injury, characterized by slight epithelial loosening and/or mild separation of the lamina propria or submucosa; 2 indicated moderate injury, characterized by evident villus shortening, moderate lamina propria/submucosal separation, edema, and inflammatory cell infiltration; 3 indicated severe injury, characterized by marked disruption of mucosal architecture, severe edema, villus sloughing, and prominent inflammatory infiltration; and 4 indicated extensive villus loss, mucosal necrosis, or severe structural destruction. The final score for each sample was calculated as the average score from randomly selected fields.

### Immunohistochemistry and immunofluorescence

Immunohistochemistry (IHC) was carried out on 5-μm paraffin sections as previously described [56]. Briefly, after deparaffinization and rehydration, tissue sections were incubated with primary antibodies against GPX4 (1:1,000), 4-HNE (1:5,000), CD45 (1:1,000), F4/80 (1:4,000), and SLC7A11 (1:50) overnight at 4 °C. A horseradish peroxidase (HRP)-conjugated secondary antibody (1:500) was then applied at room temperature for 1 h. For visualization, diaminobenzidine (DAB) substrate was applied, and the sections were counterstained with hematoxylin. IHC staining was evaluated under light microscopy (Olympus IX71, Olympus Corporation), and the degree of immune cell infiltration was quantified by analyzing CD45- and F4/80-positive cells. For immunofluorescence staining, sections were probed overnight at 4 °C with antibodies to ZO-1 (1:200) and MUC2 (1:200) and then incubated in the dark with fluorophore-conjugated secondary antibodies for 1 h at room temperature. 4′,6-Diamidino-2-phenylindole (DAPI) was used for nuclear counterstaining. Images were captured using a fluorescence microscope (Eclipse C1, Nikon, Japan). Scale bars are indicated in the figures. For IHC quantification of GPX4, 4-HNE, CD45, F4/80, and SLC7A11, the positive staining area was quantified using ImageJ and expressed as the percentage of positively stained area relative to the total tissue area. For immunofluorescence analysis of ZO-1 and MUC2, fluorescence intensity was quantified from at least 3 randomly selected fields per section using ImageJ. All image analyses were performed in a blinded manner.

### Biochemical measurement

CAT activity, SOD activity, and MDA content were quantified with commercial assay kits. GSH content and T-AOC were measured using the corresponding assay kits. Total iron and ferrous iron (Fe^2+^) concentrations in tissue and cell samples were measured using iron and ferrous ion assay kits, respectively.

### Assessment of intracellular oxidant levels using 2′,7′-dichlorofluorescein diacetate

Intracellular oxidant levels were monitored with a Reactive Oxygen Species Assay Kit according to the kit instructions. For this assay, IPEC-J2 cells were seeded into 6-well plates at 1 × 10^6^ cells per well. After the indicated treatments, IPEC-J2 cells were loaded with 2′,7′-dichlorofluorescein diacetate (DCFH-DA) for 20 min and rinsed 3 times in PBS. Fluorescence micrographs were finally acquired on an inverted fluorescence microscope.

### Assessment of lipid peroxidation by BODIPY 581/591 C11 staining

Lipid peroxidation in IPEC-J2 cells was assessed using BODIPY 581/591 C11 staining. For confocal microscopy analysis, IPEC-J2 cells were seeded onto glass coverslips in 6-well plates at 1 × 10^5^ cells per well and cultured overnight. After the indicated treatments, cells were incubated with 2.5 μM BODIPY 581/591 C11 in serum-free DMEM/F12 for 30 min at 37 °C in the dark. Unbound probe was removed by washing the cells 3 times with PBS. After a PBS rinse, coverslips were mounted on slides with DAPI-containing Antifade Mounting Media to counterstain nuclei. Confocal images were acquired with the same settings across all groups. Oxidized BODIPY C11 was detected at 488-nm excitation/500- to 550-nm emission, reduced BODIPY C11 at 559-nm excitation/580- to 630-nm emission, and DAPI at 405-nm excitation. Oxidized BODIPY fluorescence intensity was measured in at least 3 randomly selected fields per sample using ImageJ and normalized to the mean value of the control group.

For flow cytometric analysis, IPEC-J2 cells were plated at 1 × 10^6^ cells per well and treated as described above. After treatment, cells were stained with 2.5 μM BODIPY 581/591 C11 in serum-free DMEM/F12 at 37 °C for 30 min in the dark and washed twice with PBS. Cells were harvested by trypsinization, resuspended in PBS, and analyzed immediately using a BD FACSVerse flow cytometer (BD Biosciences, USA). Oxidized BODIPY C11 was read in the fluorescein isothiocyanate (FITC) channel (excitation 488 nm, emission 527/32 nm), with at least 10,000 viable cells acquired per sample. Flow cytometry data were analyzed with FlowJo software (version 10.8, BD Biosciences, USA). Lipid peroxidation was quantified as the mean fluorescence intensity (MFI) of oxidized BODIPY C11 in the FITC channel.

### Transmission electron microscopy

Following treatment, IPEC-J2 cells were fixed with 2.5% glutaraldehyde for 24 h at 4 °C and subsequently postfixed in 1% osmium tetroxide. The specimens were dehydrated through graded ethanol and acetone, embedded in resin, and cut into ultrathin sections. After uranyl acetate and lead citrate staining, mitochondrial morphology was examined at 120 kV using a Tecnai Spirit Bio-TWIN transmission electron microscope.

### RNA extraction and qPCR

qPCR was conducted based on a previously reported procedure [57]. Total RNA was extracted from cells and tissues using TRIzol reagent. cDNA was synthesized using a PrimeScript RT kit. qPCR was performed with gene-specific primers and SYBR Green chemistry on a StepOnePlus system. For mouse jejunal tissues, *Actb* was used as the internal reference gene, whereas *GAPDH* was used for IPEC-J2 cells. Relative transcript abundance was calculated using the 2^−ΔΔCt^ method. All primer sequences are provided in Table S2.

### Western blot analysis

Western blotting followed the method described in Ref. [57]. Cells and tissues were lysed in radioimmunoprecipitation assay (RIPA) buffer, and protein concentration was determined by bicinchoninic acid (BCA) assay. Protein samples, 30 μg per lane, were separated by sodium dodecyl sulfate–polyacrylamide gel electrophoresis (SDS-PAGE) and transferred to polyvinylidene difluoride (PVDF) membranes. The membranes were incubated overnight at 4 °C with primary antibodies against ZO-1 (1:1,000), Occludin (1:2,000), GPX4 (1:1,000), SLC7A11 (1:1,000), ACSL4 (1:5,000), and β-actin (1:2,000). After an HRP-conjugated secondary antibody, bands were developed with an enhanced chemiluminescence (ECL) system. Band density was measured with ImageJ. Target protein abundance was normalized against β-actin.

### siRNA transfection

SLC7A11-targeting siRNAs and a nontargeting siControl were used for knockdown experiments. siRNA sequences are provided in Table S2.

Cells were transfected with siRNA targeting *SLC7A11* (siSLC7A11) or negative control siRNA (siControl) using Lipo 8000 according to the manufacturer’s protocol. In brief, cells were grown in 6-well plates and transfected at 60% to 70% confluence. Six hours after transfection, the medium was replaced with fresh complete medium, and cells were maintained for an additional 24 h before RA and Erastin treatment. Knockdown efficiency was verified by qPCR and/or Western blotting.

### 16S rRNA sequencing and analysis

Microbial DNA was extracted from fecal samples. The V3–V4 region of the 16S rRNA gene was amplified using the primers 341F (5′-CCTACGGGNGGCWGCAG-3′) and 805R (5′-GACTACHVGGGTATCTAATCC-3′). Purified amplicons were indexed and subjected to Illumina MiSeq sequencing. Sequencing data were processed using QIIME2 (version 2024) to analyze microbial diversity and community structure. Alpha-diversity was assessed using the Simpson index, and beta-diversity was analyzed using PCoA. Microbial taxonomic profiles were summarized at both the phylum and genus levels. Differential taxa among groups were screened using linear discriminant analysis effect size (LEfSe).

### Metagenomic DNA extraction, sequencing, and analysis

Fecal microbial genomic DNA was extracted for metagenomic sequencing. Metagenomic libraries were prepared and sequenced in paired-end mode on a NovaSeq 6000 platform. Alpha-diversity was evaluated using the Chao index in QIIME2 (version 2024). Beta-diversity was computed from Bray–Curtis distances, with PCoA used to display intergroup differences in community composition. The PCoA plots and associated marginal distributions were generated using R (version 4.0.3). Phylum-level abundance matrices were used to infer microbial co-occurrence networks. Taxon–taxon associations were estimated using Spearman correlation. Correlations with |ρ| > 0.6 and *P* < 0.01 after multiple-testing correction were retained for network construction. Networks were visualized using Gephi (version 0.10.1), and node colors represent phylum-level taxonomic classification. Global and group-specific networks were generated separately to assess ASTA- and DEX-associated ecological remodeling.

KEGG pathway enrichment was analyzed using annotated metagenomic reads to determine metabolic pathways that differed among groups. Pathway analysis was performed using Diamond (version 0.8.35). To identify key microbial taxa contributing to retinol metabolism, differential abundance and contribution analyses were integrated. LEfSe was used to identify discriminative taxa, and taxa with significant enrichment and high pathway contribution were considered candidate retinol metabolism-associated bacteria. The effect size, statistical significance, and relative contribution of these taxa were visualized as bar plots.

### Nontargeted metabolomic profiling

Six independent bacterial culture samples were analyzed per group by Shanghai Biotree Biomedical Technology Co. Ltd. A 100-μl aliquot of each sample was mixed with 400 μl of methanol/acetonitrile (1:1, v/v) containing isotope-labeled internal standards. After sonication and incubation at −40 °C for 1 h, the extracts were centrifuged at 13,800*g* for 15 min at 4 °C. The resulting supernatants were subjected to LC-MS/MS, and pooled samples were included for quality control.

Separation was performed on a Vanquish UHPLC system equipped with an ACQUITY UPLC BEH Amide column (2.1 × 50 mm, 1.7 μm) and coupled to an Orbitrap Exploris 120 mass spectrometer. Mobile phase A was water containing 25 mM ammonium acetate and 25 mM ammonium hydroxide, and mobile phase B was acetonitrile. The gradient was 95% B at 0 to 0.25 min, 95% to 65% B at 0.25 to 3.50 min, 65% to 40% B at 3.50 to 4.00 min, 40% B at 4.00 to 4.50 min, 40% to 95% B at 4.50 to 4.55 min, and 95% B at 4.55 to 6.00 min. The flow rate was 0.50 ml/min, the column temperature was 30 °C, and the injection volume was 2 μl.

Data were acquired in positive and negative ion modes over mass/charge ratio (*m*/*z*) 70 to 1,200. The main parameters were as follows: spray voltage, 3.8/−3.4 kV; sheath gas, 50 arbitrary units; auxiliary gas, 15 arbitrary units; capillary temperature, 320 °C; full-scan resolution, 60,000; MS/MS resolution, 15,000; and normalized collision energies, 20, 30, and 40. Raw files were converted using ProteoWizard and processed with an in-house R package. Metabolites were annotated against BiotreeDB version 3.0 using retention time, precursor mass, and MS/MS spectra. Differential metabolites were identified using variable importance in projection (VIP) > 1 and *P* < 0.05.

### Targeted fecal metabolomic profiling

Targeted metabolomic profiling of fecal samples was performed by Shanghai Biotree Biomedical Technology Co. Ltd. using 5 biological replicates per group. Frozen samples were extracted with precooled methanol/water (4:1, v/v) containing internal standards, followed by homogenization, sonication, incubation at −40 °C, and centrifugation at 13,800*g* for 15 min at 4 °C.

Metabolites were analyzed using an Agilent 1290 Infinity II UHPLC system coupled to a SCIEX Triple Quad 6500+ mass spectrometer in multiple reaction monitoring mode. Reversed-phase and hydrophilic interaction separations were performed using ACQUITY UPLC BEH C18 and Atlantis Premier BEH Z-HILIC columns, respectively. The source parameters were as follows: curtain gas, 35 psi; ion-spray voltage, +5,000/−4,500 V; source temperature, 400 °C; and ion-source gases 1 and 2, 50 psi. Metabolites were quantified against authentic standards using internal-standard calibration curves and expressed as nmol/g feces.

### Molecular docking and molecular dynamics simulation

Molecular docking and molecular dynamics (MD) simulation were performed to evaluate the potential interaction between RA and SLC7A11. The 3-dimensional structure of SLC7A11 was obtained from the Protein Data Bank (PDB ID: 7P9U), and the structure of RA was retrieved from PubChem. Protein and ligand structures were prepared using standard procedures, including removal of water molecules, hydrogen addition, and energy minimization. Docking was run in AutoDock Vina (version 1.1.2), and the lowest-energy pose was taken as the starting conformation for MD. Structural visualization was carried out using PyMOL (version 2.5.0) and Discovery Studio Visualizer (version 2024).

MD simulation was conducted using GROMACS (version 2024.1). The protein–ligand complex was solvated in an explicit water box, neutralized with counterions, and subjected to energy minimization followed by equilibration under constant number of particles, volume, and temperature (NVT) and constant number of particles, pressure, and temperature (NPT) conditions. A production simulation was then performed for 100 ns. Complex stability was evaluated using root mean square deviation (RMSD), whereas residue-level flexibility was assessed using root mean square fluctuation (RMSF).

### Bacterial genome sequencing and analysis

The strains *L. muris, M. gordoncarteri,* and *M. intestinale* were inoculated into chopped meat carbohydrate (CMC) medium, anaerobically cultured at 37 °C for 72 h, and then centrifuged at 12,000 rpm and 4 °C for 10 min. The supernatant was discarded, and genomic DNA was extracted for whole-genome analysis. Subsequent high-throughput sequencing was commissioned to Shanghai Majorbio Bio-pharm Technology Co. Ltd. (Shanghai, China). Library construction and sequencing were performed using both second-generation (Illumina NovaSeq) and third-generation (PacBio Sequel) sequencing platforms. Retinol metabolism-related genes were annotated by aligning against 6 major databases: COG, NR, SWISS-PROT, PFAM, KEGG, and GO. The annotated data were visualized using Circos v0.69 for genome circular mapping, and functional annotations were analyzed on the Majorbio Cloud Platform.

### Bacterial culture and in vitro metabolism of ASTA

*M. gordoncarteri*, *L. muris*, and *M. intestinale* were used in this study. Each strain was initially cultured anaerobically in CMC medium at 37 °C for 48 h to establish optimal growth conditions.

For the in vitro metabolism assay, equal amounts of bacterial suspension (1 × 10^7^ colony-forming units/ml) were incubated with ASTA at a final concentration of 15 μg/ml for 24 h under strain-matched culture conditions. Vehicle-treated bacterial cultures served as controls. In parallel, ASTA-containing medium without bacteria was included as a nonbiological control to exclude spontaneous degradation of ASTA during incubation. After treatment, cultures were immediately placed on ice and centrifuged at 4,000*g* for 10 min at 4 °C to separate bacterial pellets from culture supernatants. Both fractions were collected for downstream metabolite extraction.

### Statistical analysis

At least 3 biological replicates were included in each experiment. Data are shown as mean ± SEM. Group differences were evaluated by one-way analysis of variance (ANOVA) followed by Tukey’s post hoc test. *P* < 0.05 was considered significant. GraphPad Prism was used for statistical analysis.

## Ethical Approval

All experimental animal procedures used in this experiment were approved by the Animal Care and Use Committee of Zhejiang University and complied with institutional guidelines (ZJU20250579).

## Data Availability

The 16S rRNA, metagenomic, and whole-genome sequencing data have been deposited in the NCBI Sequence Read Archive (SRA) under the accession numbers PRJNA1344425, PRJNA1347062, and PRJNA1345402, respectively. The metabolomics data are available in the EMBL-EBI MetaboLights database under the identifier MTBLS13140.
