# Supplementary material for: Astaxanthin Alleviates Intestinal Ferroptosis through Gut Commensal Lepagella muris-Mediated Retinoic Acid Production and SLC7A11 Activation
Source: Research (Wash D C). 2026 Sep 2;9:1421. doi: 10.34133/research.1421 (PMC13534808; doi:10.34133/research.1421)
Supplement: Supplementary file 1 [file research.1421.f1.docx]

**Supplementary Figure**

**Supplementary Figure 1.** (A) Volcano plot showing the effects of 201 FDA-approved small-molecule drugs on RUNX2 activity. (B) Volcano plot showing the effects of 20 FDA-approved small-molecule drugs on NF-κB activity. (C) Violin plot showing the effects of 4 FDA-approved small-molecule drugs on ALP activity. (D) The viability of BMSCs was determined using a CCK8 reagent with different concentrations of DAB and incubation times. (E) Mice were administered daily oral gavage: the treatment groups received different concentrations of DAB dissolved in a mixed solvent consisting of dimethyl sulfoxide (DMSO) and F12 medium, while the control group received an equivalent volume of vehicle solvent matched for DMSO and F12 concentrations and the internal organs were stained with HE after 60 days (scale bar: 100 μm). (F) Serum biochemical analysis of mice after 60 days of DAB treatment. Key parameters reflecting liver function (ALT, AST), kidney function (CRE, BUN), and metabolic profile (GLU) are shown. For in vivo experiments, data are presented as mean ± SD (n = 6 per group). For in vitro experiments, data are presented as mean ± SD (n = 5 per group). One-way ANOVA was used. Statistical significance: *P<0.05, **P<0.01, ***P<0.001, ****P<0.0001; ns, not significant.

**Supplementary Figure 2.** (A) Quantification of western blot results in Figure 2B. (B) Quantification of western blot results in Figure 2I. (C) Quantification of western blot results in Figure 2J. Data are presented as mean ± SD (n = 5 per group). One-way ANOVA was used. Statistical significance: *P<0.05, **P<0.01, ***P<0.001, ****P<0.0001; ns, not significant.

**Supplementary Figure 3.** (A) qRT-PCR analysis of autophagy-related genes (*SQSTM1, Map1lc3a, Map1lc3b, Pink1, TSC1, Ulk1*) in BMSCs treated with different concentrations of DAB. (B) Quantification of western blot results in Figure 3E-G. (C) Quantification of western blot results in Figure 3I. (D) Quantification of western blot results in Figure 3N. (E) Quantification of western blot results in Figure 3O. (F) Quantification of western blot results in Figure 3P. (G) Quantification of western blot results in Figure 3Q. (H) Quantification of western blot results in Figure 3S–T. (I) Quantification of western blot results in Figure 3U. (J) Compound C inhibits AMPK pathway activation in BMSCs, as determined by qRT-PCR analysis of osteogenic markers (*RUNX2, OSX*). (K) Quantitative analysis of ALP-positive area in Figure 3V. (L) Quantitative micro-CT parameters in Figure 3W: BMD, BV/TV, Tb.Th, Tb.N, Tb.Sp. For in vivo experiments, data are presented as mean ± SD (n = 6 per group). For in vitro experiments, data are presented as mean ± SD (n = 5 per group). One-way ANOVA was used. Statistical significance: *P<0.05, **P<0.01, ***P<0.001, ****P<0.0001; ns, not significant.

**Supplementary Figure 4.** (A) DARTS assays showing DAB exerted no protective effect on PRKAA1, PRKAA2, PRKAB2, PRKAG1, PRKAG2, PRKAG3. (B) CETSA assays showing DAB exerted no protective effect on PRKAA1, PRKAA2, PRKAB2, PRKAG1, PRKAG2, PRKAG3.

**Supplementary Figure 5.** (A) Validation of PRKAB1 knockdown efficiency in BMSCs by western blot. (B) qRT-PCR analysis of osteogenic markers (*RUNX2, OSX, OPN*) in PRKAB1-knockdown or NC BMSCs. (C–D) Western blot analysis of osteogenic markers (RUNX2, OSX, OPN) (C) and quantification (D) in PRKAB1-knockdown or NC BMSCs. (E–F) ALP staining (E, scale bar: 200 μm) in PRKAB1-knockdown or NC BMSCs and quantification of ALP-positive area (F). Data are presented as mean ± SD (n = 5 per group). One-way ANOVA was used. Statistical significance: *P<0.05, **P<0.01, ***P<0.001, ****P<0.0001; ns, not significant.

**Supplementary Figure 6.** (A-B) Western blot of AMPK activation and inhibition following treatment with AICAR and Compound C in PRKAB1 mutant or wild-type PRKAB1 BMSCs (A) and quantification (B). (C–D) Western blot of AMPK and mTORC1 activity under DAB treatment in PRKAB1 mutant or wild-type PRKAB1 BMSCs western blot (C) and quantification (D). Data are presented as mean ± SD (n = 5 per group). One-way ANOVA was used. Statistical significance: *P<0.05, **P<0.01, ***P<0.001, ****P<0.0001; ns, not significant.

**Supplementary Figure 7.** (A–C) qRT-PCR of osteoclastogenic markers (*ACP5, CTSK*) (A) and western blot (B) in BMDMs treated with Compound C and DAB under RANKL stimulation and quantification (C). (D–F) qRT-PCR of osteoclastogenic markers (*ACP5, CTSK*) (D) and western blot (E) in PRKAB1 mutant or wild-type PRKAB1 BMDMs treated with DAB under RANKL stimulation and quantification (F). Data are presented as mean ± SD (n = 5 per group). One-way ANOVA was used. Statistical significance: *P<0.05, **P<0.01, ***P<0.001, ****P<0.0001; ns, not significant.

**Supplementary Figure 8.** (A–B) Western blot analysis of RELA phosphorylation (A) and quantification (B) in RELA mutant or wild-type RELA RAW264.7 cells treated with DAB and TNF-α. (C–D) Western blot analysis of inflammatory markers (COX2, iNOS) (C) and quantification (D–E) in RELA mutant or wild-type RELA RAW264.7 cells treated with DAB and TNF-α. (F) Validation of PRKAB1 knockdown efficiency in BMDMs by western blot. (G–K) qRT-PCR analysis of osteoclastogenic markers (*c-Fos, NFATc1, MMP9, ACP5, CTSK*) in RELA-knockdown or NC BMDMs. (L–Q) Western blot of osteoclastogenic markers (c-Fos, NFATc1, MMP9, ACP5, CTSK) (L) and quantification (M–Q) in RELA-knockdown or NC BMDMs. (R–S) TRAP staining (R, scale bar: 200 μm) in RELA-knockdown or NC BMDMs, and quantification of the number and area of TRAP-positive multinucleated cells (MNCs, nuclei ≥ 3) (S). Data are presented as mean ± SD (n = 5 per group). One-way ANOVA was used. Statistical significance: *P<0.05, **P<0.01, ***P<0.001, ****P<0.0001; ns, not significant.

**Supplementary Figure 9.** Graphical abstract of the proposed mechanism underlying dabigatran‑mediated balance of osteoclastogenesis and osteoblastogenesis in osteoporosis by targeting RELA and PRKAB1.
